# *In Vitro* Antioxidative Evaluation of **α**- and **β**-Carotene, Isolated from Crude Palm Oil

**DOI:** 10.1155/2013/351671

**Published:** 2013-11-12

**Authors:** Surashree Sen Gupta, Mahua Ghosh

**Affiliations:** ^1^Department of Chemical Technology, University of Calcutta, Kolkata, West Bengal 700 009, India; ^2^Department of Chemical Technology, University College of Science & Technology, University of Calcutta, 92 A.P.C. Road, Kolkata 700009, India

## Abstract

The present work describes the isolation of **α**- and **β**-carotene from crude palm oil and their antioxidant potential in an *in vitro* model. Pure product was isolated by the method adopted. Antioxidant activities of the isolated **α**- and **β**-carotene were analyzed in five different concentrations of 0.001, 0.005, 0.01, 0.05, and 0.1% (w/v). From the several assays conducted, an observation was made that the antioxidant activity of the product shifted between antioxidant and prooxidant effects depending on the concentration and the system analyzed. The metal chelation, DPPH radical scavenging, and superoxide scavenging activities showed almost similar results in terms of high activity at lowest concentrations. ABTS-scavenging activity was displayed only by a particular antioxidant concentration of 0.1%. Lipid peroxidation assay pronounced the activity of 0.1% antioxidant in inhibiting oxidation of sensitive bioactive lipids. *In vitro* antidenaturation test again specified the efficacy of low concentrations in preventing protein denaturation. Through this study a definite dosage formulation for consumption of carotenoids is being proposed which will enhance health promotion and prevent chronic diseases when taken as fortified foods or dietary supplements.

## 1. Introduction

*α*- and *β*-carotene are important members of the carotenoid family. As a retinol precursor with a high conversion rate, *α*- and *β*-carotene provide a substantial proportion of the vitamin A in the human diet [1]. Recently some researchers have shown that *α*- and *β*-carotene, due to their antioxidant activities, possess important health-promoting activities which might be helpful in the prevention and protection against a number of serious health disorders such as cancer, cardiovascular disease, and colorectal adenomas [2]. For these reasons, there is a strong interest in using *α*- and *β*-carotene and other carotenoids as functional ingredients in food products. 

Crude palm oil has a significant amount of carotene that can be extracted and, in recent years, various methods for extracting carotenes from palm oil have been developed. Of these, the method involving the combination of trans-esterification and molecular distillation processes is the most cost-effective one with ease of execution [3], and this was followed in this study.

A number of studies have shown that *α*- and *β*-carotene and other carotenoids have lipid-soluble antioxidant activity. In homogeneous lipid solutions, in membrane models, and also in intact cells, *α*- and *β*-carotene have been mostly studied [4, 5]. Recent findings suggest that the position and orientation of the carotenoids in the membrane are important factors in determining their relative effectiveness in protecting against free radicals [6]. *α*- and *β*-carotene partially or completely protect intact cells (e.g., human liver cell line HepG2) against oxidant-induced lipid peroxidation, and the protective effect is independent of provitamin A activity [4]. *α*- and *β*-carotene suppressed lipid peroxidation in mouse and rat tissues [5]. The consumption of food-based antioxidants like *α*- and *β*-carotene seems to be useful for the prevention of macular degeneration and cataracts [7]. A well-known fact is that *α*- and *β*-carotene quench singlet oxygen with higher efficiency than many other antioxidants [8].

In contrast to the physiologically relevant properties, the knowledge on antioxidant potential of *α*- and *β*-carotene, *in vitro*, is scarce as most studies conducted so far were *in vivo*. Hence, the aim of the present study was to isolate *α*- and *β*-carotene from crude palm oil, their characterization, and *in vitro* investigation of their antioxidative efficacy.

## 2. Experimental

### 2.1. Materials

Crude palm oil imported from Indonesia was collected from local Vanaspati manufacturer. All other reagents were of analytical grade and procured from Merck India Ltd., Mumbai. India.

### 2.2. Isolation of *α*- and *β*-Carotene from Crude Palm Oil

Crude palm oil was chemically transesterified with methanol and sodium hydroxide. Oil and alcohol were taken in different molar ratios and stirred while maintaining a high temperature for a definite time interval [3]. Syntheses of esters were monitored by thin layer chromatography. The effects of different reaction parameters for this particular reaction, such as temperature, substrate molar ratio, and concentration of alkali, were optimized with individual sets of reaction mixture. Oil : methanol ratio of 1 : 10 on weight basis, stirring rate of 200 rpm, temperature of 80°C for 3 hours, and a concentration of 0.2% NaOH were optimised for maximum yield of *α*- and *β*-carotene. A two-phase mixture comprising a glycerol phase and an ester phase consisting of fatty esters and the carotene was obtained. The ester phase was distilled at 300°C at 10^−01 ^mbar pressure in a molecular distillation unit (SIBATA, Japan, serial no. N40394). The residue was diluted 5 times and saponified with ethanolic potassium hydroxide at 80°C. The carotene was extracted from the saponified residue with a mixture of hexane and water where the extract phase was rich in carotene. Pure carotene was obtained on solvent (hexane) removal which was crystallized and recrystallized with acetone.

### 2.3. Spectrophotometric Analysis

For spectrophotometric analysis 0.01 g measured amount of the crystallized carotene product was taken in a calibrated test tube and dissolved into 25 mL of petroleum ether. The solution was scanned under UV-visible spectrophotometer (UV-1700 PharmaSpec, UV-VIS Spectrophotometer, Shimadzu, Japan) between 300–600 nm. The *λ*_max⁡_ value was noted and the molar extinction coefficient (*ε*_max⁡_) using 1 cm cell was calculated for definite concentration of the sample. Further spectral conformation was carried out by scanning the solid carotene crystals under the UV-visible range of 200–800 nm with UV spectrophotometer (U-3501, Hitachi, Japan).

### 2.4. Characterisation by Thin Layer Chromatography (TLC)

Crystalline samples were dissolved in petroleum ether and spotted in glass TLC plates coated with silica gel stationary phase. The TLC plate was placed in a chamber saturated with solvent vapour. The solvent system consisted of 60% petroleum ether/20% acetone/20% dichloromethane [9]. After development of the plates, they were visualized by subjecting them to iodine vapour. The *R*_*f*_ values for the spot on the TLC plate were calculated:

(1) $$\begin{array}{c} R_{f}=\frac{\text{distance}\;\text{from}\;\text{baseline}\;\text{to}\;\text{sample}\;\text{front}}{\text{distance}\;\text{from}\;\text{baseline}\;\text{to}\;\text{solvent}\;\text{front}}. \end{array}$$

### 2.5. Characterisation by High Performance Liquid Chromatography (HPLC)

Analysis of the carotenoids was conducted using Waters HPLC (Milford, Massachusetts, US) with a Waters 1525 binary HPLC pump consisting of a vacuum degasser and a Waters 2487 dual *λ* absorbance detector. Reverse-phase HPLC equipped with Nova-pak C_18_ column, 3.9 × 150 mm, 4 *μ*m was used for the analysis of the antioxidant. The UV detector was set at 450 nm. The mobile phase consisted of acetonitrile : methanol : tetrahydrofuran (50 : 45 : 5 by volume) [10]. The crystallized carotenoids were dissolved in 5 mL of the mobile phase. The mobile phase was isocratic solvent. Injection volume of 200 *μ*L and the flow rate were set at 1 mL/min. Data acquisition was completed in 40 minutes.

### 2.6. Antioxidant Assay of Isolated Carotene Crystals

The antioxidant activity of the isolated carotenoids was examined by seven different *in vitro *assay systems. Different concentrations of the antioxidant were prepared in ethanol. The concentrations were 0.001, 0.005, 0.01, 0.05, and 0.1% (w/v). Their antioxidant activities were measured consecutively.

#### 2.6.1. Assay of DPPH Radical-Scavenging Activity

Antioxidant activity of *α*- and *β*-carotene was determined by the scavenging activity of the stable DPPH free radical. The method was described by Katerere and Eloff [11]. Different concentrations of the test samples were placed into test tubes taking 0.2 mL from each of them with 4 mL of 0.2 mM of ethanolic solution of DPPH. Absorbance at 517 nm was determined after 40 minutes using a solution of ethanol and DPPH (3 : 1) as control. Radical scavenging activity was expressed as the inhibition percentage and was calculated using the following formulae:

(2) $$\begin{array}{c} \%\;\text{Radical}\;\text{scavenging}\;\text{activity}=\left[\frac{A_{0}-A_{1}}{A_{0}}\right]\times 100, \end{array}$$

where *A*_0_ is the absorbance of the control and *A*_1_ is the absorbance of the sample.

#### 2.6.2. Assay of Reductive Potential

The reductive potential of the antioxidant was determined according to the method of Dorman and Hiltunen [12]. The reaction mixture containing different concentrations of the antioxidants (50–250 *μ*g/mL) in 1 mL of ethanol, phosphate buffer (2.5 mL, 0.2 M, pH 6.6), and potassium ferricyanide (2.5 mL, 1% wt/vol) was incubated at 50°C for 20 minutes. A portion (2.5 mL) of trichloroacetic acid (10% wt/vol) was added to the mixture, which was then allowed to rest for 10 minutes. From the mixture 2.5 mL was taken and mixed with distilled water (2.5 mL) and FeCl_3_ (0.5 mL, 0.1% wt/vol), and the absorbance was measured at 700 nm in a spectrophotometer. Higher absorbance of the reaction mixture indicated greater reductive potential.

#### 2.6.3. Assay of Metal Chelating Activity

The Fe^2+^ chelating ability of antioxidants was estimated by the method of Dinis et al. [13]. Samples with concentrations of about 50–250 *μ*g/mL were added to 0.08 mL of FeCl_2_ (2.5 mmol/L) solution. To the system 0.3 mL of ferrozine (6 mmol/L) solution was added and the mixture was shaken vigorously and left to stand at room temperature for 10 minutes. Absorbance was thereby measured spectrophotometrically at 562 nm. Percentage of inhibition of ferrozine-Fe^2+^ complex formation was calculated as follows:

(3) $$\begin{array}{c} \%\;\text{inhibition}=\left[\frac{A_{0}-A_{1}}{A_{0}}\right]\times 100, \end{array}$$

where *A*_0_ is the absorbance of the control and *A*_1_ is the absorbance of the sample.

#### 2.6.4. ABTS Radical Scavenging Activity

ABTS radical cation scavenging activity of flaxseed lignan was assessed by the method of Re et al. [14] and Zhao et al. [15] with some minor modifications. Measured volume of 5 mL of 7 mM ABTS was mixed with 88 *μ*L of 140 mM K_2_S_2_O_8_ and kept overnight. Next day the solution was diluted with 50% ethanol and an initial absorbance of 0.7 was set at 734 nm. Then 1 mL of diluted ABTS was mixed with 20 *μ*L of sample and absorbance was measured at 734 nm at 1-second intervals for 5 minutes.

#### 2.6.5. Lipid Oxidation in a Linoleic Acid Emulsion Model System

The ammonium thiocyanate method was used to assess the antioxidant activity of flaxseed lignans with some minor modifications of the procedure of Gülçin [16]. A linoleic acid preemulsion was made by vortexing 0.28 g of linoleic acid with 0.28 g of Tween 20 in 50 mL of 0.05 *μ*L phosphate buffer (pH 7.4). Working solutions of SDG (0.2 mL) were added and mixed with 2.5 mL linoleic acid emulsion and 2.3 mL phosphate buffer (0.2 *μ*L), vortexed, and incubated at 37°C overnight. Aliquots (100 *μ*L) from the incubated mixture were withdrawn at 1, 24, 48, 72, 96, and 120 hours of incubation and tested for lipid peroxidation by adding 5 mL of ethanol (75%), 0.1 mL of NH_4_SCN (30% w/v), and 0.1 mL of FeCl_2_ (0.1% w/v). The absorbance of the reaction mixture was measured at 500 nm against ethanol

(4) $$\begin{array}{c} \%\;\text{inhibition}=\left[\frac{A_{0}-A_{1}}{A_{0}}\right]\times 100, \end{array}$$

where *A*_0_ is the absorbance of the control and *A*_1_ is the absorbance of the sample.

#### 2.6.6. Superoxide Anion Scavenging Activity

Superoxide anion scavenging activity of flaxseed lignan was based on the method described by Yaping et al. [17] and Wang et al. [18] with some minor modifications. About 4.5 mL of Tris-HCl buffer (50 mmol/L, pH 8.2) and 1.0 mL tested samples with various concentrations were mixed in tubes with lids. Then the mixture was incubated for 20 min in the water bath at 25°C. Meanwhile, 0.4 mL of 25 mmol/L pyrogallol preheated at 25°C was added immediately. After 4 min, the reaction was terminated by 0.1 mL HCl solution (8 mol/L) and the mixture was centrifuged at 4000 rpm for 15 min. The absorbance of sample and control was determined by UV spectrophotometer at 325 nm. Scavenging activity was calculated using the following equation:

(5) Superoxide  anion  scavenging  activity (%)=(A0−As)A0×100,

where *A*0 is the absorbance without sample and *As* is absorbance with sample.

#### 2.6.7. Assay of *In Vitro* Anti-denaturation Effects

The assay helps to assess the anti-denaturation/anti-inflammatory effect of proteins. The method is based on the works of Williams [19]. An amount of 2.5 mL 1% BSA was mixed with 2.5 mL tris acetate buffer (0.05 M) and 2.5 mL of the test solutions. The mixtures were heated at 69°C for 4 minutes and cooled and then the absorbances of the turbidities were read at 660 nm

(6) $$\begin{array}{c} \%\;\text{inhibition}\;\text{of}\;\text{denaturation}=\frac{\left(A0-As\right)}{A0}\times 100, \end{array}$$

where *A*0 is the absorbance of control and *As* is absorbance with sample.

### 2.7. Statistical Analysis

Statistical analysis was performed with one-way analysis of variance (ANOVA). When ANOVA detected significant differences between mean values, means were compared using Tukey's test. For statistical studies OriginLab software (Origin7, OriginLab Corporation, Northampton, UK) was used. Statistical significance was designated as *P* < 0.05. Three replications for each of the experiments and assays were conducted (*n* = 3). A mean of the three values was reported in each case. The values are expressed as Mean ± SEM.

## 3. Results and Discussions

### 3.1. Isolation, Spectroscopic Analysis, and TLC of *β*-Carotene

**Figure 1:**
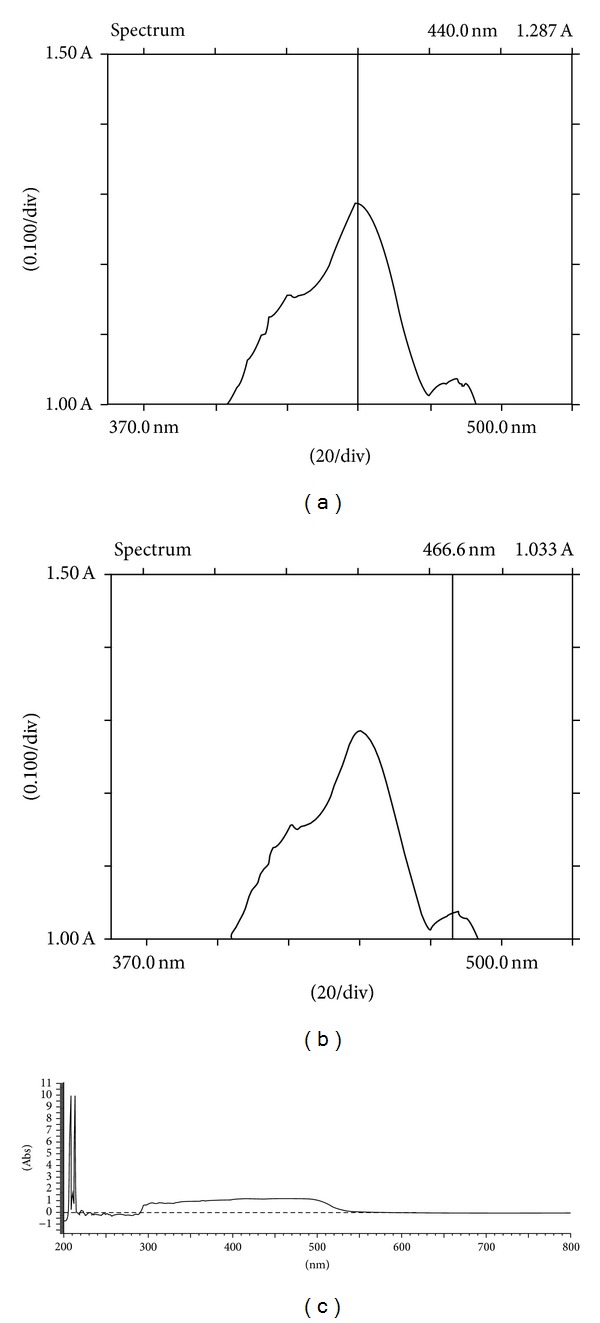
Absorbance spectrum of *β*-carotene/*α*-carotene pigments isolated from crude palm oil: (a) absorbance maxima of *β*-carotene at 440 nm; (b) absorbance of *α*-carotene at 466.6 nm; (c) absorbance profile of *β*-carotene/*α*-carotene mixture in a crystalline form.

In the present work the palm oil was initially transesterified and then concentrated by distillation for isolating *α*- and *β*-carotene. Amount of the carotene crystals obtained was 1.2%. Spectroscopic studies showed maximum absorption at 440 nm (Figure 1(a)) and a minor absorbance peak at 466.6 nm (Figure 1(b)). The peak at 440 nm corresponds to the absorbance by *β*-carotene, as confirmed by calculation of the molar extinction coefficient (*ε*_max⁡_)

(7) $$\begin{array}{c} \varepsilon_{\max}=\frac{A}{CL}, \end{array}$$

where *A* is absorbance of the specified molecule at maximum wavelength (*λ*_max⁡_), *C* is concentration of the active molecule, and *L* is distance (1 cm).

The extinction coefficient was calculated to be 1, 28, 300 L mol^−1^ cm^−1^ which is in correlation with established values of extinction coefficient [20]. The minor peak at 466.6 nm is that of *α*-carotene which is in correlation with the extinction coefficient (1, 02, 979 L mol^−1^ cm^−1^) of *α*-carotene [21]. 

The absorption at given wavelength range can be expressed as the sum of individual carotenoids as observed in Figure 1(c), where UV/visible spectrophotometric study of the crystalline carotenoid pigment displays a uniform plateau-shaped absorption range representing an assemblage of *α*- and *β*-carotene. The range was observed between 300 to 550 nm which is in harmony with previously derived results. 

On thin layer chromatographic analysis *R*_*f*_ factor was calculated to be 0.95 which corresponds to previously reported values [22]. The single spot indicated that *α*- and *β*-carotene had overlapped over the specified area resulting in the incurrence of an intermediate value of retention factor.

### 3.2. Detection of *β*-Carotene Using High Performance Liquid Chromatography (HPLC)

**Figure 2:**
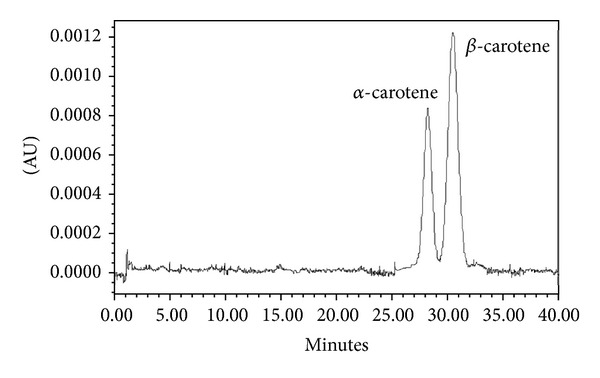
HPLC chromatogram of *α*- and *β*-carotene.

On HPLC two peaks were observed with retention times at 28.2 and 30.5 minutes. *β*-carotene eluted as a single peak at 30.5 minutes (Figure 2) and *α*-carotene at 28.2 minutes, as deciphered by comparison with authentic standards.

### 3.3. Antioxidant Assay of Isolated *β*-Carotene

The antioxidant activity of the isolated carotene crystals were examined by seven different *in vitro *assay systems. Different concentrations of the antioxidant was prepared in ethanol. The concentrations prepared were 0.001, 0.005, 0.01, 0.05, and 0.1% (w/v). Their antioxidant activities were measured in terms of DPPH radical scavenging activity, reducing activity, metal chelation, ABTS radical scavenging activity, lipid peroxidation, superoxide scavenging activity, and anti-denaturation effect.

#### 3.3.1. Assay of DPPH Radical-Scavenging Activity

**Figure 3:**
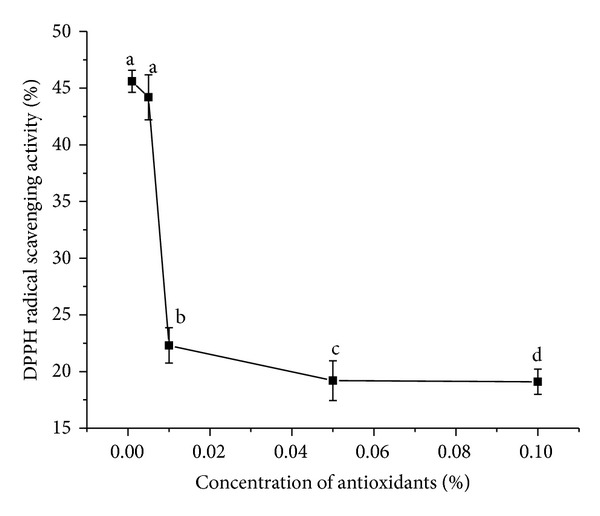
DPPH radical scavenging activity for *α*- and *β*-carotene crystals at different concentrations. Values are Mean ± SEM of 3 observations. At the 0.05 level, difference in superscripts (a, b, c, d) indicates significant difference in means.

The DPPH radical scavenging method was based on the reduction of methanolic DPPH solution in the presence of a hydrogen donating antioxidant, due to the formation of the non-radical form DPPH-H [23]. Monitoring the decrease of the radical in terms of decreasing absorbance values leads to the assessment of the antioxidant activity of the product. The carotene crystals were able to reduce and decolourise 1,1-diphenyl-2-picrylhydrazyl efficiently at low concentrations, via their hydrogen donating ability (Figure 3). This is an electron transfer-based assay which measures the capacity of an antioxidant in the reduction of an oxidant. Here the degree of colour change is correlated with the sample's antioxidant activity. 

The order of antioxidant potency by DPPH method is conclusive of the behavior of the antioxidant at different concentration gradients. Colour interference by *α*-carotene with DPPH chromogen and *β*-carotene may result in a lower measured DPPH activity at high concentrations [24]. Moreover the presence of higher concentrations of antioxidants results in prooxidant effects which induce oxidative stress, usually by inhibiting antioxidant systems. Hence the lowest concentration of the antioxidant (0.001%) that was analysed for DPPH radical scavenging activity was found to be the optimum concentration that showed efficacy in scavenging the free radicals. Furthermore, on comparing the mean values, all the concentration means varied significantly among themselves except for 0.001% and 0.005% concentrations at *P* < 0.05.

#### 3.3.2. Assay of Reductive Potential

**Figure 4:**
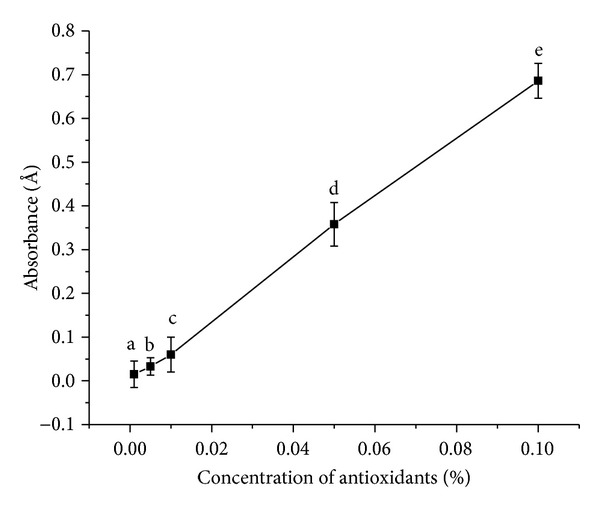
Reducing activity of *α*- and *β*-carotene crystals at different concentrations. Values are Mean ± SEM of 3 observations. At the 0.05 level, the difference in superscripts (a, b, c, d, e) indicates significant difference in means.

Again assay of reducing capacity is an effective means to understand the antioxidant activity of various antioxidants. Reducing capacity serves as a significant indicator of the potential antioxidant activity of any bioactive species. Here reduction potential bears a proportional dependency on the absorbance measured (Figure 4). Here all the different concentration mean values were significantly different from each other at *P* < 0.05. The measured absorbance serves to indicate the change in reduction potential of the tested species. The reducing power (transformation of Fe^3+^ to Fe^2+^) of the antioxidant makes it an efficient electron donor, which can react with free radicals to convert them to more stable products, thereby terminating radical chain reactions. The antioxidant exerts an antioxidant effect by reducing Fe^3+^ to Fe^2+^. Such bioactive property of the antioxidant leads to the development of its chemoprotective potential too. From this assay it was deduced that in terms of reducing activity *α*- and *β*-carotene showed the expected tendency of concentration variation. With increasing carotenoid concentrations the absorbance increased indicating an increase in the reducing activity of the antioxidant. Hence higher amounts of the antioxidant will serve as an efficient reducing agent, though its radical scavenging activity showed a contrary behaviour.

#### 3.3.3. Assay of Metal Chelating Activity

**Figure 5:**
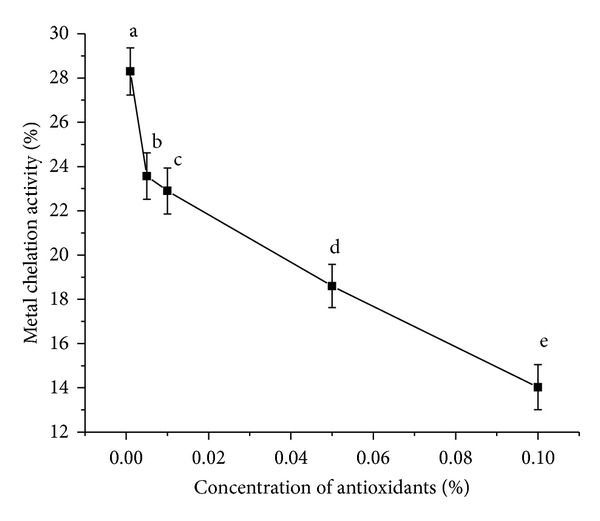
Metal chelation activity of *α*- and *β*-carotene crystals at different concentrations. Values are Mean ± SEM of 3 observations. At the 0.05 level, the difference in superscripts (a, b, c, d, e) indicates significant difference in means.

Again metal chelation activity is an example of a complexation reaction where Ferrozine [disodium salt of 3-(2-pyridyl)-5,6-bis(4-phenylsulfonic acid)-1,2,4-triazine] is a complex-forming agent of Fe(II) and will form a magenta complex Fe(II)-(Ferrozine)(III) with maximum absorbance at 562 nm. Hence in the presence of a reducing agent the complex formation is hampered resulting in decrease in the colour of the complex and hence a decrease in the absorbance. Measurement of absorbance therefore allows estimating the metal chelating activity of the coexisting chelator [25]. In this case *α*- and *β*-carotene interfered with the formation of ferrous-ferrozine complex. The steric hindrance generated by the bulky carbon chains of *α*- and *β*-carotene prevented the unsaturated groups from acting as chelating agents at higher concentrations. Moreover with increasing concentrations the prooxidant effect is active and *α*- and *β*-carotene become the source of complexation, resulting in higher absorbance values and hence reduced metal chelation activities (Figure 5). In fine, *α*- and *β*-carotene act as a better DPPH-radical scavenger than a metal chelator. The presence of unsaturated groups could be a contributing factor towards their effective reducing property. All the different concentration mean values were significantly different from each other at *P* < 0.05.

#### 3.3.4. ABTS Radical Scavenging Activity

**Figure 6:**
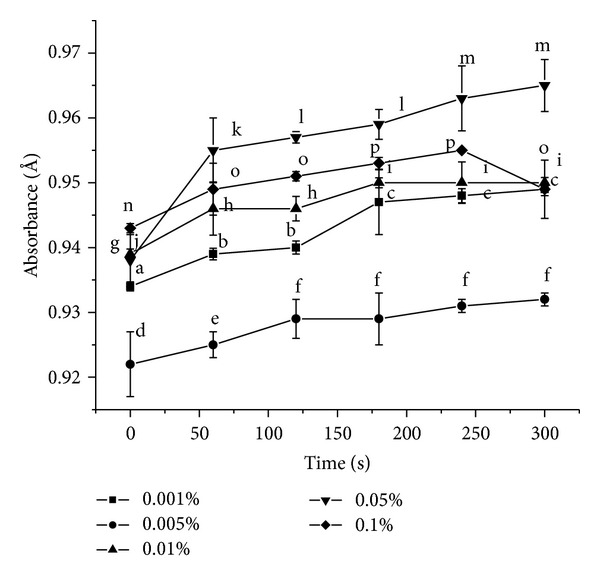
ABTS-radical scavenging activity of *α*- and *β*-carotene crystals at different concentrations. Values are Mean ± SEM of 3 observations. At the 0.05 level, at time intervals 0, 60, 120, 180, 240, and 300 seconds, the difference in superscripts indicates significant difference in means. Superscripts (1) (a, b, c) for 0.001%; (2) (d, e, f) for 0.005%; (3) (g, h, i) for 0.01%; (4) (j, k, l, m) for 0.05%; and (5) (n, o, p) for 0.1% are used.

The ABTS assay is based on the ability of antioxidants to scavenge the long-life radical cation ABTS^+^. The scavenging of radicals produces a decreased absorbance at 734 nm. Here absorbance was found to increase at 734 nm for all concentrations, except for 0.1% concentration (Figure 6). In case of 0.1% concentration, commencement of radical-scavenging activity was observed beyond 240 seconds, as indicated by lowering absorbance values. 

Unlike DPPH radical scavenging activity, the ABTS-scavenging power was exhibited only at 0.1% antioxidant concentration. Apparently here the antioxidant activity of *α*- and *β*-carotene cannot be well differentiated based on their concentrations as can be established in the case of DPPH method or metal chelation. Generation of oxidative stress was evident from the increase in absorbance values at 0.05% concentration. The lowest concentration of 0.001% showed higher absorbance readings than the ones at 0.005% concentration. This could be due to the inability of the antioxidant to scavenge the long-life free radicals at the minimal concentration. At still higher value of 0.01% concentration, the absorbance value reaches an almost constant value beyond 4 minutes indicating a possibility of ABTS-scavenging capacity at higher time periods. At *P* < 0.05, for concentrations 0.001%, 0.005%, 0.01%, 0.05%, and 0.1% the population means were significantly different from each other throughout the time range tested except for: 60, 120 seconds and 180, 240, 300 seconds among themselves for 0.001% concentration; 120, 180, 240, 300 seconds among themselves for 0.005% concentration; 60, 120 seconds and 180, 240, 300 seconds among themselves for 0.01% concentration; 120, 180 seconds and 240, 300 seconds among themselves for 0.05% concentration; 60, 120, 300 seconds and 180, 240 seconds among themselves for 0.1% concentration. 

#### 3.3.5. Lipid Oxidation in a Linoleic Acid Emulsion Model System

**Figure 7:**
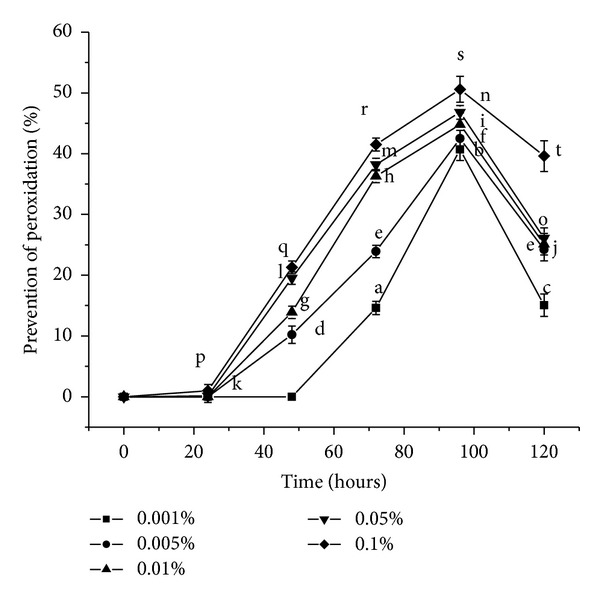
Prevention of lipid peroxidation by *α*- and *β*-carotene crystals at different concentrations. Values are Mean ± S.E.M of 3 observations. At the 0.05 level, for the time span 0 hours, 24 hours, 48 hours, 72 hours, 96 hours, and 120 hours, the difference in superscripts indicates significant difference in means. Superscripts (1) (a, b, c) for 0.001%; (2) (d, e, f) for 0.005%; (3) (g, h, i, j) for 0.01%; (4) (k, l, m, n, o) for 0.05%; and (5) (p, q, r, s, t) for 0.1% are used.

The relative inhibitory effect of *α*- and *β*-carotene on lipid peroxidation using a model linoleic acid peroxidation assay is shown in Figure 7. The basis of lipid peroxidation method involves the use of heat as the free radical initiator which instigates the oxidation of Fe^2+^ to Fe^3+^ (8) and is consequently measured by assessing the amount of peroxyl radicals that is generated in the prepared linoleic acid emulsion, due to the lipid oxidation reaction of the linoleic acid [26]

(8) $$\begin{array}{c} {\text{Fe}}^{2+}+\text{LOOH}={\text{Fe}}^{3+}+{\text{LO}}^{●}+{\text{OH}}^{-}, \end{array}$$

where LOOH denotes a lipid derived hydroperoxide and LO^●^ denotes a lipid peroxyl radical. The Fe^3+^ subsequently reacts with ammonium thiocyanate to form ferric thiocyanate. The absorbance at 500 nm measures the amount of ferric thiocyanate formed in the medium as a result of the above reaction. Higher absorbance readings (i.e., lower prevention of lipid peroxidation) are associated with increased concentration of peroxyl radicals in the solution which occurs simultaneously with the formation of the colouring complex. *α*- and *β*-carotene served to scavenge the Fe^2+^ ion from the medium, where the scavenging efficacy increased with increasing concentration of the antioxidants. A variety of beta carotene isomers and metabolites were used to test for various *in vitro* assays like FRAP assay, lipid peroxidation, and so forth. No ferric reducing activity (FRAP assay) was observed for the isomers. Between the major isomers no significant differences in bleaching the ABTS^●+^ or in scavenging peroxyl radicals (ROO^●^) generated by thermal degradation of AAPH (using a chemiluminescence assay) were detected [27]. The carotenoids displayed lipophilic antioxidant activity by dissolving completely in the linoleic acid emulsion and serving as an efficient radical scavenger. A mixture of *α*- and *β*-carotene in association increased the activity of the product crystals against lipid peroxidation as indicated by high level of inhibition of lipid peroxidation and therefore sufficient antioxidant activity at higher concentrations.

In case of 0.001% concentration prevention of peroxidation was not detected before 72 hours unlike 0.1% concentration where 0.97% prevention was observed only after 24 hours. Beyond 4 days the inhibition of lipid peroxidation reduced for all concentrations. Thus antioxidant activity of the products showed potency maximum up to 4 days, beyond which the effectiveness diminished gradually and predominance of the peroxyl radicals was observed, as indicated by higher absorbance values. At *P* < 0.05, for the time span 0 hours, 24 hours, 48 hours, 72 hours, 96 hours, and 120 hours the population means are significantly different from each other for all the five different carotene concentrations except for: 72 from 120 hours for 0.005% concentration.

#### 3.3.6. Superoxide Anion Scavenging Assay

**Figure 8:**
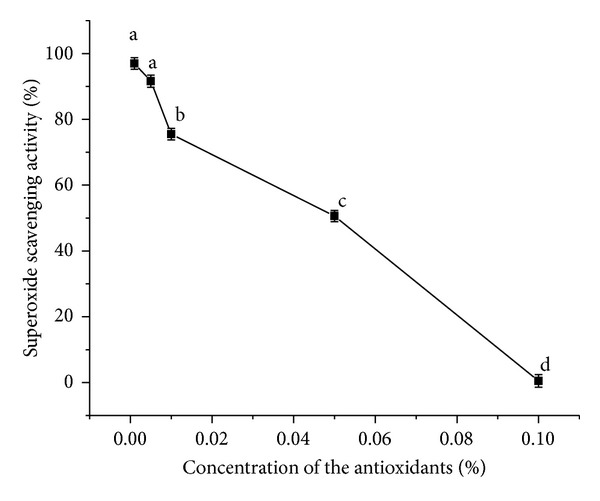
Superoxide scavenging activity by *α*- and *β*-carotene crystals at different concentrations. Values are Mean ± SEM of 3 observations. At the 0.05 level, the difference in superscripts (a, b, c, d) indicates significant difference in means.

Again *α*- and *β*-carotene crystals exhibited a concentration-dependent superoxide scavenging activity similar to DPPH radical scavenging activity. The losses of linearity in the dose response curve at low concentrations of *α*- and *β*-carotene were a reflection of not only their superoxide scavenging efficiency, but also the prooxidant effect of the products. As both *α*- and *β*-carotene can interchange between a reduced form and an oxidised form, they display antioxidant and pro-oxidant properties related to their dosage and half-life [28]. Necessary preponderance must be given to the antioxidant activity of carotenoids at low concentrations, especially while considering their scavenging activities (Figure 8). Here pyrogallol acted as the free radical initiator which absorbed oxygen from the air; turning purple from a colourless solution, by forming superoxide anions. Superoxide anion is an oxygen-centered reactive oxygen species (superoxide anion and hydrogen peroxide are the main reactive oxygen species causing the oxidation of cells and tissues). They react with protons in water solution to form hydrogen peroxide (9), which serve as a substrate for the generation of hydroxyl radicals and singlet oxygen [29]

(9) $$\begin{array}{c} 2{{\text{O}}_{2}}^{-}+2{\text{H}}^{+}\to {\text{H}}_{2}{\text{O}}_{2}+{\text{O}}_{2}. \end{array}$$

Superoxide radical is a very harmful species to cellular components as a precursor of more reactive oxygen species and is known to be produced *in vivo* and can result in the formation of H_2_O_2_ via dismutation reaction. H_2_O_2_ is a nonradical reactive oxygen species which acts as a strong oxidant leading to harmful reactions. Here *α*- and *β*-carotene at a concentration of 0.001%, scavenged superoxide anions, with utmost efficiency, thereby reducing the probability of peroxide formation. Radical scavengers can be prooxidant unless linked to a radical sink, and superoxides are such radical sinks and hence efficiently scavenge radicals at low concentrations. Increasing concentrations of the antioxidants lead to the appearance of prooxidant activity resulting in the decrease in scavenging capacity. Here the antioxidant itself behaved as a radical that bigoted another radical with higher efficiency at lower concentrations and it promoted oxidation at higher concentrations. At *P* < 0.05, all the population means are significantly different from each other except for 0.001% and 0.005% concentrations. 

#### 3.3.7. Assay of *In Vitro* Antidenaturation Effects

**Figure 9:**
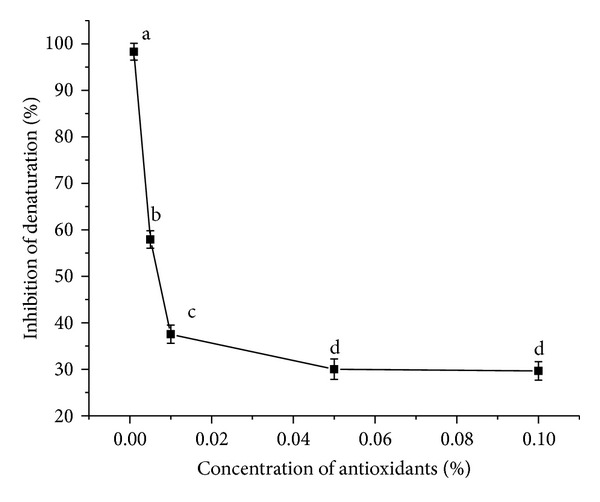
Inhibition of denaturation *in vitro* by *α*- and *β*-carotene crystals at different concentrations. Values are Mean ± SEM of 3 observations. At the 0.05 level, the difference in superscripts (a, b, c, d) indicates significant difference in means.

The present study is the first of its kind to report the efficacy of *β*-carotene in protecting cells from denaturation/inflammatory effect *in vitro* (Figure 9). 

When BSA is heated it undergoes denaturation and expresses antigens associated to Type III hypersensitive reaction which are related to diseases such as serum sickness, glomerulonephritis, rheumatoid arthritis, and systemic lupus erythematosus. An in-depth study of the antidenaturation effect of *α*- and *β*-carotene can be utilised for the treatment of such diseases by their specific dosage formulations. An efficient antioxidant is one which is able to compete with endogenous scavengers and interact with endogenous pathways by localizing themselves in the appropriate area. One of the features of several nonsteroidal anti-inflammatory drugs is their ability to stabilize (prevent denaturation) heat treated BSA at pathological pH [pH 6.2–6.5] [19]. The increased synthesis of heat shock results in a ubiquitous physiological response of cells to environmental stress and hence promotes degradation of proteins. In this study an attempt was made *in vitro* to study the course of action of *α*- and *β*-carotene in preventing thermal denaturation and aggregation of BSA protein at increased temperatures. A variety of cellular internal and external stress are generated due to environmental imbalance which leads to the formation of reactive oxygen species resulting in the origin of several disturbances in the normal redox conditions of the cells leading to their deterioration in the course of time. These stresses denature proteins enhancing the probability of the formation of reactive oxygen species in the medium, leading to inflammation and other physiological malfunctions. These aggregates, if not disposed of or their formation being prevented, can lead to cell death. In response to the appearance of damaged proteins, cells induce the expression of such harmful species. However as an effective antioxidant *α*- and *β*-carotene prevented such denaturation, which functioned as molecular chaperone and prevented protein aggregation or degradation. Carotenoids (including *β*-carotene) were found to promote health when taken at dietary levels, but showed adverse effects when consumed at a high dose by subjects who smoke or were exposed to asbestos [30]. The effectiveness was the greatest at low concentrations, which can be utilized in drug designing. The high apparent antioxidant capacity of *α*- and *β*-carotene at low concentrations also has an added advantage in terms of cost-effectiveness of the developed drug product. At *P* < 0.05, the concentration means are significantly different from each other except for the concentrations 0.05% and 0.1%.

## 4. Conclusion

From the results obtained, both *α*- and *β*-carotene were isolated effectively and their antioxidant study gave interesting observations.  Table 1 summarizes the efficacy of the carotenoids in responding to the different antioxidant assays. While 0.001% concentration was effective DPPH/superoxide radical scavenger, metal chelator, and antidenaturant, 0.1% concentration showed effective reducing activity and lipid peroxidation. Moreover 0.1% was the only concentration which showed any ABTS radical scavenging activity. Compounds often enhance the antioxidant capacity of cells but are ineffective in test tube assays. The antioxidant activity of *α*- and *β*-carotene can shift into a prooxidant phase, depending on such factors as oxygen tension or carotenoid concentration. Good correlations observed among the different hydrophilic antioxidant assays, namely, metal chelation, DPPH radical, and superoxide scavenging activities, suggested that these methods have almost similar predictive capacity for antioxidant activities of various concentrations of *α*- and *β*-carotene. ABTS-scavenging activity was displayed at a particular antioxidant concentration of 0.1%. In case of lipophilic antioxidant assay it was observed that almost 51% inhibition of lipid peroxidation was displayed by 0.1% activity. The right dose essentially differentiates a potent remedy from a subsequent harmful intake. Hence in hydrophilic environment mostly lower concentrations of antioxidants were effective, whereas in lipophilic medium higher concentrations were effective. This could be due to the higher lipid affinity of the carotenoids, being nonpolar in nature. In consequence they can act as substantial chemoprotectants and prevent harmful physiological activities, if consumed in a proper dose.

## Figures and Tables

**Table 1:** Relative efficacy of each antioxidant assay for individual antioxidant concentration.

Concentrations (%)	Antioxidant assay						
	DPPH radical scavenging activity (%)	Reducing activity (Å)	Metal chelation (%)	ABTS radical scavenging activity (%)	Lipid peroxidation (%)	Superoxide radical scavenging activity (%)	Anti-denaturation assay (%)
0.001	+++++	+	+++++	−	+	+++++	+++++
0.005	++++	++	++++	−	++	++++	++++
0.01	+++	+++	+++	−	+++	+++	+++
0.05	++	++++	++	−	++++	++	++
0.1	+	+++++	+	+	+++++	+	+
